# Supplementary figures and images for: Genomic characteristics and clinical significance of CD56+ circulating tumor cells in small cell lung cancer
Source: Sci Rep. 2023 Mar 3;13:3626. doi: 10.1038/s41598-023-30536-9 (PMC9984363; doi:10.1038/s41598-023-30536-9)

Supplemental Fig1

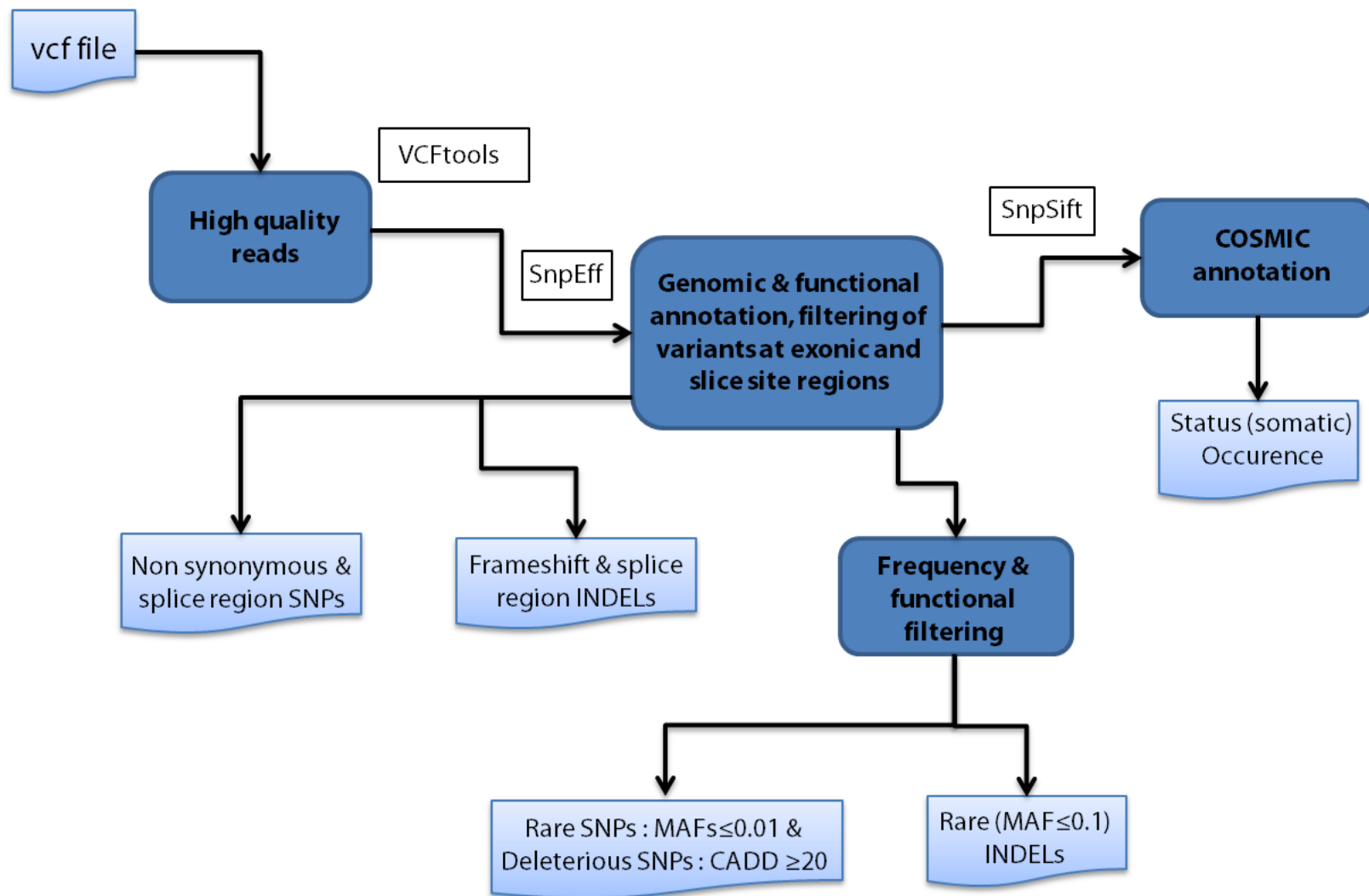

Supplement: Supplementary file 2 — Supplementary Figure S1. [file 41598_2023_30536_MOESM2_ESM.pdf]

# Supplemental Fig2

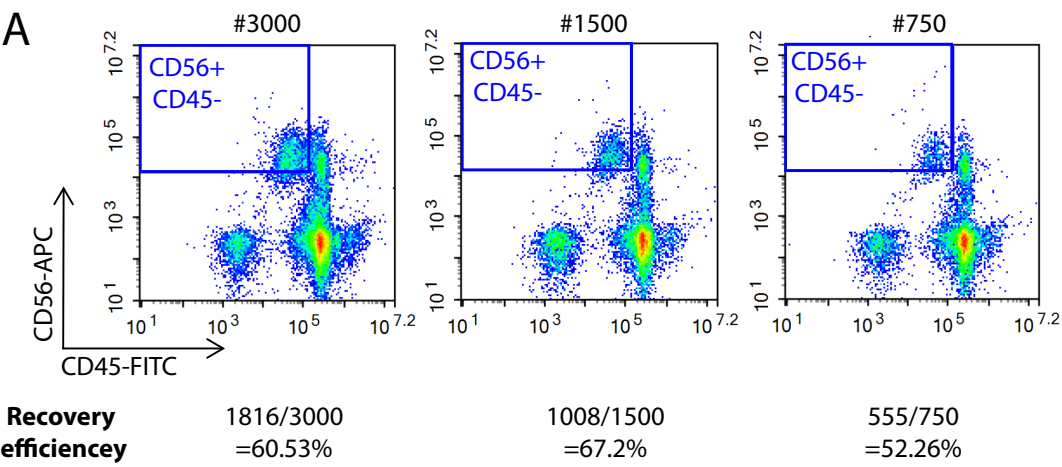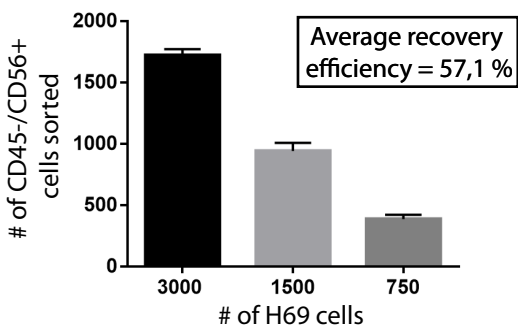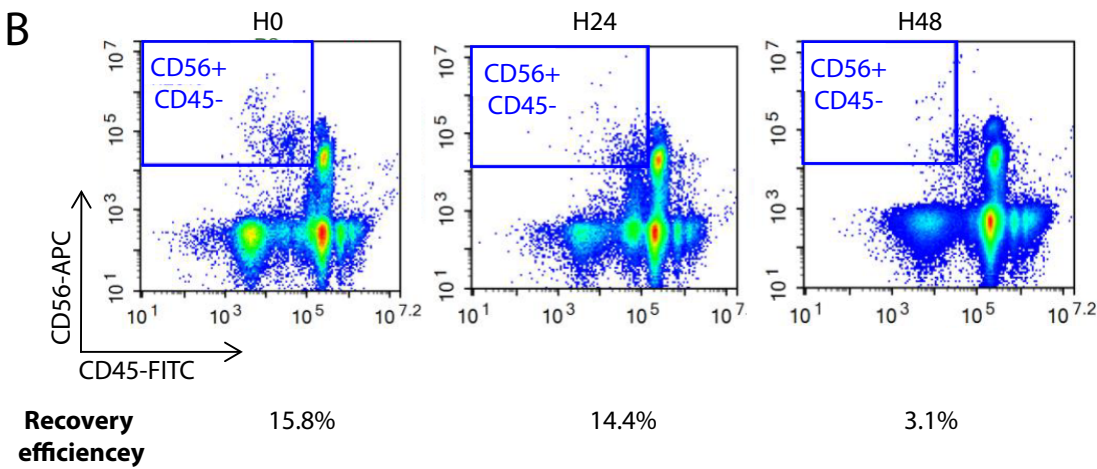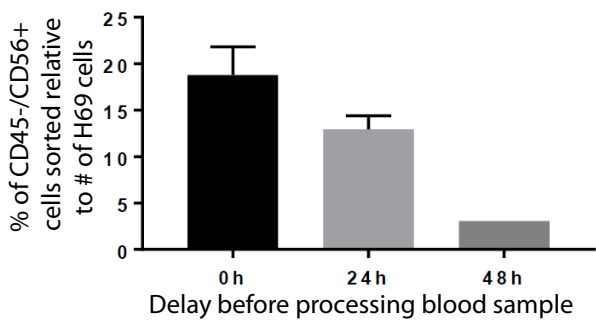

Supplement: Supplementary file 3 — Supplementary Figure S2. [file 41598_2023_30536_MOESM3_ESM.pdf]

## Supplemental Fig 4

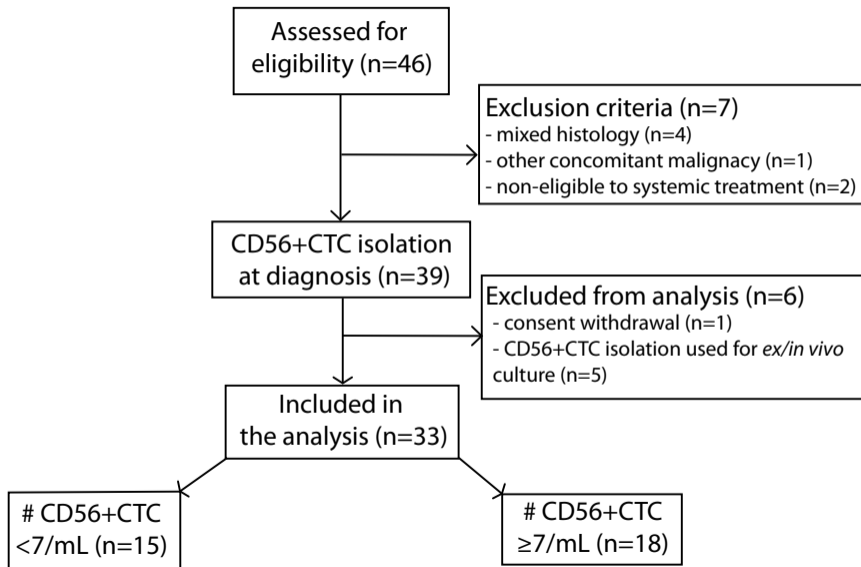

Supplement: Supplementary file 5 — Supplementary Figure S4. [file 41598_2023_30536_MOESM5_ESM.pdf]

Supplemental Fig 5

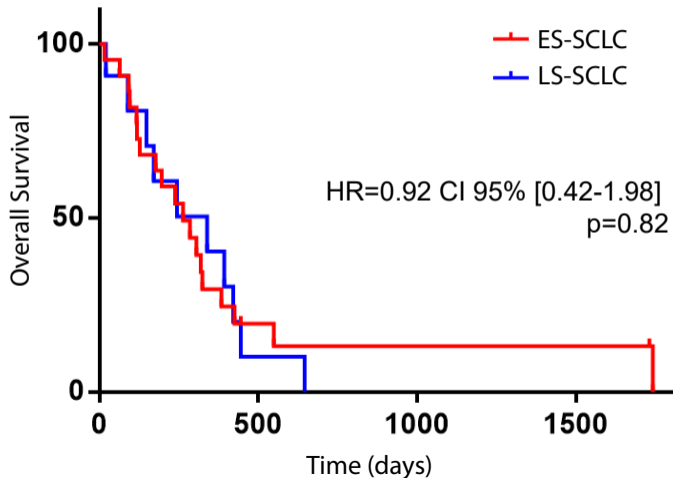

Supplement: Supplementary file 6 — Supplementary Figure S5. [file 41598_2023_30536_MOESM6_ESM.pdf]
