# Supplementary material for: Genomic characteristics and clinical significance of CD56+ circulating tumor cells in small cell lung cancer
Source: Sci Rep. 2023 Mar 3;13:3626. doi: 10.1038/s41598-023-30536-9 (PMC9984363; doi:10.1038/s41598-023-30536-9)
Supplement: Supplementary file 4 — Supplementary Figure S3. [file 41598_2023_30536_MOESM4_ESM.pdf]

Supplemental Fig3

A

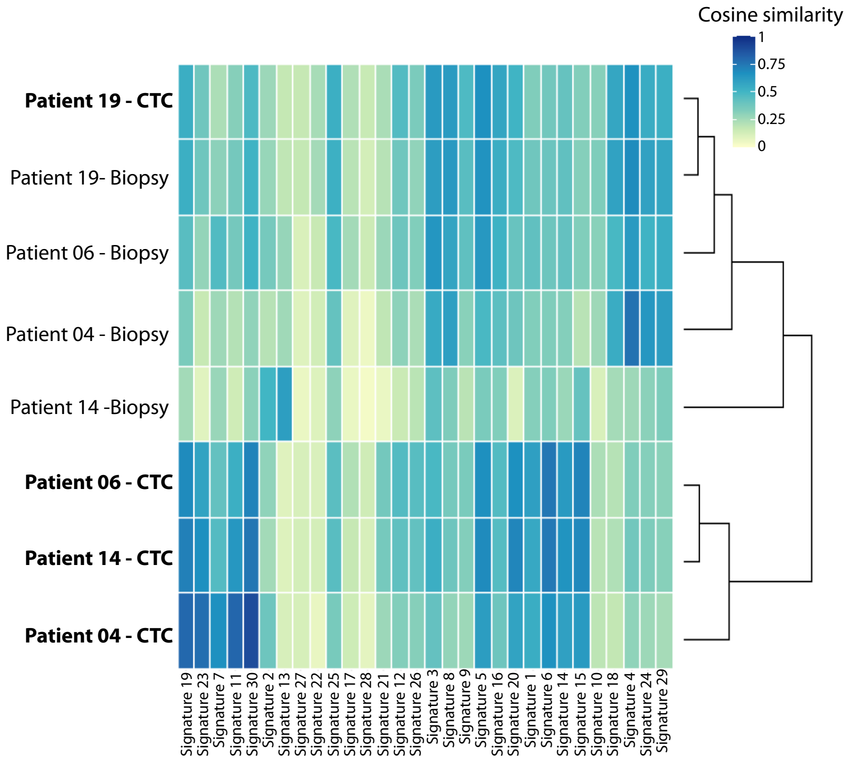

B

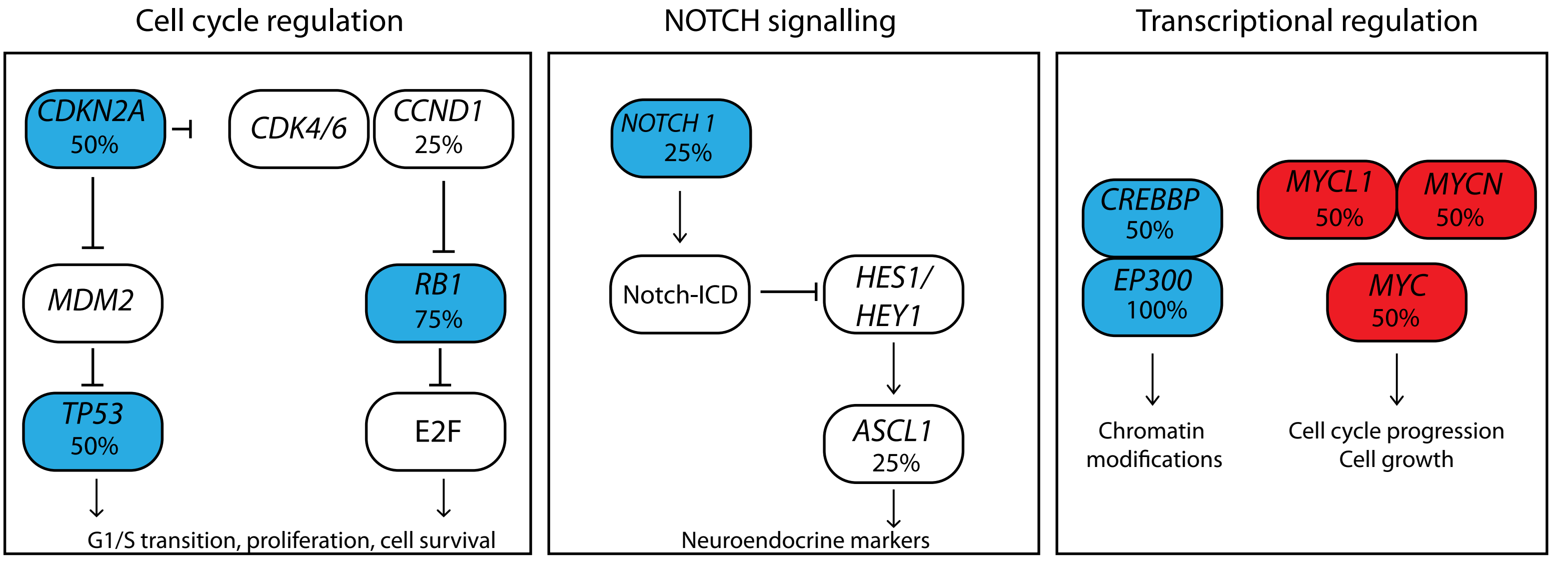

C

CD56+CTC 75 hub genes

AR	COL5A1	HCK	LRP6	PROX1
ARHGAP35	COL9A1	HDAC6	MAP3K12	PTK7
ARNT	COL9A3	HIF1A	MDM2	PTPRS
AXL	CREBBP	HIF3A	MECP2	ROCK2
BAX	CSF1	HIPK2	MED1	RUNX2
BMI1	DNM2	INSR	MEIS1	SIX4
BRAF	DSCAM	INSRR	MTOR	SMAD4
BTRC	EGLN1	ITGAV	NF1	STAT3
CACNA1A	EGR1	ITGB1	NR4A3	STAT5B
CACNA1C	EIF2AK3	JARID2	NRG1	TP53
CHD3	EP300	KALRN	PARP1	TRIM28
CITED2	EPHB1	KDM5B	PIP5K1C	TSC1
COL27A1	ERBB2	KMT2A	PLCB1	WNT1
COL4A2	ESR1	KMT2E	PRKCG	YAP1
COL4A3	FZD5	LAMC3	PRKDC	ZBTB7A

CD56+CTC 23 core hub genes

AR	ERBB2	NRG1
ARHGAP35	ESR1	PARP1
ARNT	HIF1A	SMAD4
BAX	KMT2A	STAT3
BRAF	MDM2	STAT5B
CREBBP	MTOR	TP53
DNM2	NF1	TSC1
EP300	NR4A3	

GENE NAME : common with all tissue databases

GENE NAME : common with 1 or 2 tissue databases

D

Rank	Term	Definition	Enrichment	Pvalue
1	1234158	Regulation of gene expression by Hypoxia-inducible Factor	4/11	0.0041
2	5663202	Diseases of signal transduction	12/386	0.0086
3	8864260	Transcriptional regulation by the AP-2 (TFAP2) family of transcription factors	4/38	0.014
4	6804114	TP53 Regulates Transcription of Genes Involved in G2 Cell Cycle Arrest	3/18	0.0162
5	6804760	Regulation of TP53 Activity through Methylation	3/19	0.0238
6	3108232	SUMO E3 ligases SUMOylate target proteins	7/181	0.0254
7	6803204	TP53 Regulates Transcription of Genes Involved in Cytochrome C Release	3/20	0.0295
8	2990846	SUMOylation	7/187	0.0352
9	8878159	Transcriptional regulation by RUNX3	5/96	0.0374
10	2219528	PI3K/AKT Signaling in Cancer	5/102	0.0416
11	8941855	RUNX3 regulates CDKN1A transcription	2/7	0.0464
12	1234174	Cellular response to hypoxia	4/75	0.0499
